# Variations in the Crystal Lattice of Tb-Dy-Fe Magnetostrictive Materials: The Lattice Constant Disturbance

**DOI:** 10.3390/mi14122166

**Published:** 2023-11-28

**Authors:** Jiaxin Gong, Jiheng Li, Xiaoqian Bao, Xuexu Gao

**Affiliations:** State Key Laboratory for Advanced Metals and Materials, University of Science and Technology Beijing, 30 Xue Yuan Road, Beijing 100083, China; gongjiaxin416@163.com (J.G.); lijh@ustb.edu.cn (J.L.); bxq118@ustb.edu.cn (X.B.)

**Keywords:** magnetostrictive materials, Tb-Dy-Fe alloy, crystal structure, scanning transmission electron microscopy, lattice parameter

## Abstract

In Tb-Dy-Fe alloy systems, Tb_0.29_Dy_0.71_Fe_1.95_ alloy shows giant magnetostrictive properties under low magnetic fields, thus having great potential for transducers, microsensors, and other applications. The C15 cubic crystal structure of Tb-Dy-Fe has long been thought to be the source of giant magnetostriction. It is surprising that such a highly symmetrical crystal structure exhibits such a large magnetostrictive strain. In this work, the lattice parameters of Tb_0.29_Dy_0.71_Fe_1.95_ magnetostrictive materials were studied by processing atomic-resolution images. The selected area diffraction patterns show a face-centered cubic structure, but the fast Fourier transform diagram shows that the cubic structure has obvious distortion. The lattice parameters obtained by geometric phase analysis (GPA) and Gaussian model-based fitting and calculation show that the lattice constants a, b, and c are not strictly equal, and small disturbance of the lattice constants occurs based on the cubic structure. The actual crystal structure of the Tb-Dy-Fe material is a slightly disturbed cubic structure. This variation in the crystal lattice is mainly caused by the inhomogeneous composition and may be related to the giant magnetostrictive properties of Tb-Dy-Fe alloy.

## 1. Introduction

Tb-Dy-Fe materials are widely used in transducers, sensors, actuators, and other devices because of their large magnetostrictive coefficient [1,2]. Cubic Laves phase Tb_x_Dy_1−x_Fe_2_ compounds are well known for their giant magnetostriction and low magnetocrystalline anisotropy [3]. Large magnetostriction is achieved under the condition of a low field. When x = 0.27–0.3, the magnetostriction value of the alloy can reach more than 1500 ppm, and the alloy is known as Terfenol-D [4]. This RFe_2_ (R = Tb, Dy) compound with a C15 cubic structure has a large magnetostrictive effect [5]. It is surprising that such a highly symmetrical compound exhibits such a large strain.

Tb-Dy-Fe materials are generally produced by directional solidification, and the growth orientation of the materials is generally (110), (112), or (113) [6]. Tb_1−x_Dy_x_Fe_2_ is considered to be a pseudo-binary alloy with a similar crystal structure to DyFe_2_ and TbFe_2_. Tb and Dy atoms are in the same position in the cell of Tb-Dy-Fe alloy and can be substituted for each other. TbFe_2_ has a very large saturation magnetostriction coefficient, but a weak response to low magnetic fields. After some of the Tb in TbFe2 is replaced by Dy, it becomes TB-Dy-Fe pseudo-binary alloy, which becomes very responsive to low magnetic fields. The crystal structure of Tb_x_Dy_1−x_Fe_2_ (x = 0.27–0.3) materials is considered to be a typical C15 cubic structure, and the giant magnetostriction phenomenon is believed to be generated by the cubic structure. This is due to the presence of two tetrahedral sites in the C15 structure. When excited by the external magnetic field, the energy of the C15 structure is reduced by the displacement of the two tetrahedral sites in the (111) direction of the interior. This distortion drives rhomboidal distortion, which is macroscopically manifested as a magnetostriction phenomenon. X-ray diffraction data give the lattice parameter of the C15 cubic phase as a = 7.331 Å for Tb_0.27_Dy_0.73_Fe_l.95_ [7]. M. Palit et al. studied the cubic Laves phase (C15) structure of (Tb,Dy)Fe_2_ using powder diffraction and calculated its lattice parameter to be 7.32 Å [6]. Ma Tianyu confirmed that the matrix of Tb_0.3_Dy_0.7_Fe_2_ alloy is cubic Laves phase with a MgCu2-type structure through X-ray powder diffraction analysis and calculated the lattice constant a to be 7.333 Å by using the Bragg formula [8]. The lattice constant (a) was measured by D.G. Lord et al. from the (220) diffraction trace at room temperature and found to be (7.32 ± 0.13) Å [9]. J.X. Gong calculated that the lattice constants of cubic cells in Tb_0.29_Dy_0.71_Fe_1.95_ samples were a = 7.330 Å and considered that the slight change in lattice constants had an important effect on the magnetostriction [10].

However, these crystal structure data were mostly obtained via powder X-ray diffractometry, and the difference in the crystal structure between directionally solidified solids and powders in actual use was not considered. The lattice constant calculated from X-ray diffractometry data is only an average value and does not take into account the variation in interplanar spacing represented by the diffraction peak width. Lattice constants in specific microscopic regions are rarely studied. It is well known that the room-temperature magnetostrictive strains vary in the range of (1–2) × 10^3^ ppm [11]. When the rate of change in the lattice constant is in the range of a few thousandths, it is the same order of magnitude as the magnetostrictive strain. For magnetostrictive materials, this rate of change in lattice constant needs to be paid enough attention. Quantitative measurement of strain on the nanometer scale is important for Tb-Dy-Fe alloy. It is necessary to study the subtle changes in Tb-Dy-Fe crystal structure.

Tb-Dy-Fe materials used in devices are typically directionally solidified blocks. We tested the XRD peaks of directionally solidified blocks and found that the broadening of the peaks is much larger than that of the powder state, and the broadening cannot be reduced by homogenizing heat treatment. We believe that the lattice constant of Tb-Dy-Fe materials fluctuates greatly, which is larger than that of other materials, especially non-magnetic materials.

In this paper, lattice constants are calculated via Gaussian model-based fitting of atomic-resolution photographs, and the lattice constant disturbance of Tb_0.29_Dy_0.71_Fe_1.95_ is studied. The reason for the lattice constant disturbance is also discussed.

## 2. Materials and Methods

An alloy with a nominal composition of Tb_0.29_Dy_0.71_Fe_1.95_ was prepared by the Bridgeman directional solidification process in an argon atmosphere. The nominal purity for the starting materials was 99.99 wt.% for Tb, 99.99 wt.% for Dy, and 99.99 wt.% for Fe. The molten Tb-Dy-Fe was cast into an Al_2_O_3_ mold and then moved at a speed of 4 mm per minute. That is to say, the crystal directional crystallization speed was 4 mm per minute. Tb-Dy-Fe alloy prepared at this solidification rate is generally axially 110 oriented and is a commonly used material. It is easy for residual internal stress to occur during the directional growth of alloys. To reduce residual internal stresses in the crystals, routine annealing of 950 °C for 3 h is usually employed [12]. In order to homogenize the composition and completely eliminate the macroscopic internal stress, the samples in this paper were homogenized for 6 h and cooled in a furnace. The temperature of the homogenization heat treatment was 1060 °C, and it took 2 h to rise from room temperature to 1060 °C. An argon atmosphere was maintained during the heat treatment process. The bulk sample was cut along the growth direction, and the powder was obtained by manually grinding the annealed sample in an argon atmosphere. XRD patterns were obtained with Cu-Kα radiation (with wavelength λ-Kα_1_ = 1.54059 Å) on a Rigaku (Smart Lab 9Kw, Tokyo, Japan) X-ray diffractometer. The measurements were taken at 40 kV by applying a potential current of 30 mA and a scan rate of 5° min^−1^ from 15° to 100°. A scanning transmission electron microscopy (STEM) study was carried out on an aberration-corrected JEM-ARM200F (JEOL, Tokyo, Japan) electron microscope operated at 200 kV. The JEM-ARM200F is equipped with JEOL’s unique Cold field firing gun (Cold FEG) and a new Cs corrector (ASCOR) that compensates for higher-order aberrations. The combination of the cold field gun and ASCOR enables atomic-resolution imaging not only at accelerated voltages of 200 kV but also at low voltages of 30 kV. JEM-ARM200F is a high-resolution scanning transmission electron microscope that utilizes the spherical aberration corrector of the imaging system to enable a transmission electron image (TEM) resolution of up to 110 pm. With advanced analytical capabilities, the instrument enables both atom-by-atom imaging resolution and good spatial resolution for the atom-to-atom mapping of materials. The microscope offers high stability for imaging and analysis at the subnanometer scale. The STEM images and geometric phase analysis (GPA) were analyzed using DigitalMicrograph (https://www.gatan.com/installation-instructions, accessed on 7 March 2018). Atomic-resolution photographs were processed by StatSTEM software [13] (https://github.com/quantitativeTEM/StatSTEM/releases/tag/v3.1.1.html, accessed on 25 November 2020) using the Gaussian model-based fitting method. Atomic-resolution measurements of strain and displacement were obtained using geometric phase analysis and StatSTEM software.

## 3. Results and Discussion

**Figure 1:**
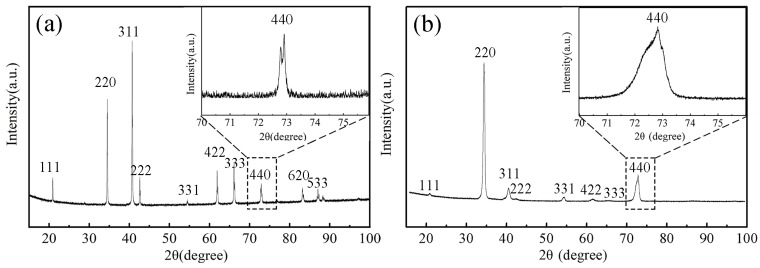
XRD patterns of the powder and directionally solidified samples of Tb_0.29_Dy_0.71_Fe_1.95_. The inset shows the profile of the 440 peak. (**a**) Powder sample. (**b**) Directionally solidified sample.

Figure 1a,b shows the XRD patterns of the powder sample and directionally solidified sample, with a typical RFe_2_ (R = Tb, Dy) Laves phase peak pattern. It can be seen that the main peak and secondary peak of the block sample are 220 and 440, respectively, and the peaks at other locations are small or hidden, which is typical of <110> preferred orientation. The error of crystal face spacing calculated by using high-angle peaks is smaller, so we focused on studying the 440 peak. The width of the 440 peak in the powder sample was only 0.5 degrees, while the 440 diffraction peak of the directionally solidified sample had a width of approximately 2 degrees, and its peak pattern was not a standard Gaussian peak pattern and was almost exactly the same shape as that reported in the literature [14,15,16]. The broadening of diffraction peaks in directionally solidified samples may be due to the superposition of diffraction peaks of two similar structures or to the large variation in the RFe_2_ phase crystal plane spacing. This paper considers the latter factor to be the main reason for peak broadening.

After taking the derivative of the Bragg equation and transforming a series of equations, the lattice strain can be estimated using the equation [17]:

(1) $$\varepsilon =|\frac{\Delta d}{d_{0}}|=|\frac{d-d_{0}}{d_{0}}|=|-cot\theta \cdot \frac{\beta }{4}|=cot\theta \cdot \frac{\beta }{4}$$

where *d*, *θ*, and β are the lattice spacing, the Bragg angle, and the width of the peak in radians, respectively. With the span of the 440 peak from 71.5° to 73.5°, the variation range of interplanar spacing *d*_440_ can be calculated to be approximately 2.4%. Therefore, the variation range of other interplanar spacings is likely to be in the same order of magnitude.

**Figure 2:**
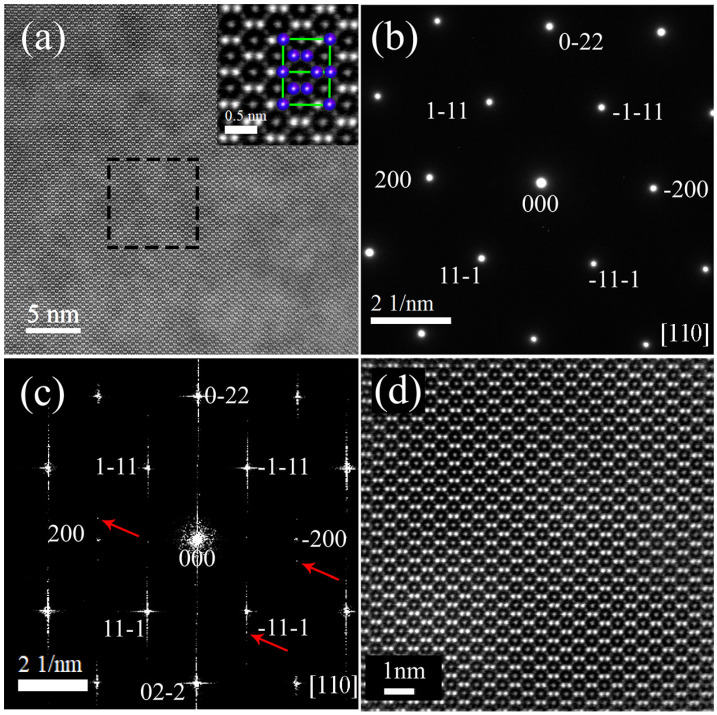
HAADF-STEM images of Tb_0.29_Dy_0.71_Fe_1.95_ alloy. (**a**) STEM image viewed from the (110) direction; the inset shows the phase relationship of a cell in an atomic image. (**b**) Selected area diffraction (SAED) pattern of the sample. (**c**) FFT of (**a**). (**d**) A typical position (black rectangle in (**a**)) was chosen for further STEM analysis.

TEM has an advantage over XRD in characterizing the crystal structure in that the structure in specific microregions can be directly observed. To accurately characterize the location of atoms, STEM was performed on the sample. Figure 2a shows an aberration-corrected scanning transmission electron microscopy high-angle annular dark field (STEM-HAADF) micrograph of Tb_0.29_Dy_0.71_Fe_1.95_, and its selected area diffraction pattern has only one set of diffraction spots, as shown in Figure 2b, indicating a single-crystal structure. The region characterized by selected area diffraction is a circular region with a diameter of approximately 1 µm and includes the region shown in Figure 2a. The spots in both the selected area diffraction pattern and the Fourier transform pattern are indexed to the 110 crystal plane of the cubic structure. Figure 2c shows the fast Fourier transform (FFT) of Figure 2a, with an area of approximately 32 nm × 32 nm, obtained with Digital Micrograph software (https://www.gatan.com/installation-instructions, accessed on 7 March 2018). Figure 2c corresponds to a frequency pattern that represents information about the periodic structure in Figure 2a. The main spots indicate that the structure of the sample has a face-centered cubic arrangement. However, the irregular shape of the main spot and the satellite spots next to it (as indicated by the red arrow) indicate widespread lattice distortion in the sample.

Since Tb (z = 65) and Dy (z = 66) have similar atomic numbers z and have similar extranuclear electron layers, their characteristic X-rays are very similar. Neither L-edge nor M-edge can accurately distinguish Tb and Dy elements. As a result, it is difficult to distinguish between Tb and Dy atoms through EDS. Fortunately, the brightness of the spot in the STEM diagram is proportional to the atomic number, and the larger the relative atomic mass, the brighter the spot, and the Tb and Dy atoms can be distinguished by this principle.

The light spots shown in Figure 2a are all Tb atoms and Dy atoms, excluding Fe atoms. The Fe atoms are not shown in the picture due to their small atomic number contrast. The atomic number of Dy is larger than that of Tb, resulting in a brighter atomic image through STEM. Tb elements with a lower atomic number are enriched in places with darker contrast, and Dy elements with a higher atomic number are enriched in places with brighter contrast. Therefore, the composition distribution (mainly referring to Tb and Dy atoms) of the sample is not uniform. Moreover, since the sample was homogenized annealed, the uneven distribution of Tb and Dy elements could not be eliminated by homogenizing annealing. The atomic number contrast in the STEM samples after 6 h of homogenization heat treatment is still obvious, indicating that long-term homogenization heat treatment can not completely eliminate the uneven distribution of Tb and Dy. A further zoom-in on the area inside the rectangle is shown in Figure 2d. The rare earth atoms (Tb and Dy) are clearly seen in the lattice of the crystal. As shown in Figure 2a,d, the sample has almost no defects, such as vacancies or interstitial atoms, and no obvious interface. The atoms are arranged in a regular uniform manner at their matrix positions, that is, there is only one set of lattices.

Geometric phase analysis (GPA) is a useful method to map strain distribution in atomically resolved scanning transmission electron microscopy (STEM) images. It is very effective for the analysis of scanning transmission electron microscopy images that resolve every atom column since it uses Fourier transforms and does not require real-space peak detection and assignment to appropriate sublattices [18]. The GPA algorithm can provide reliable and reproducible measurements of lattice strains and displacement fields with high precision in STEM images [19,20,21,22]. The accuracy of GPA depends on the choice of x and y direction and the process of Fourier filtering, the size of the reference area, the defocus value, and the diffracted beams that determine the fringe contrast. This method has high accuracy, as demonstrated by its ability to measure up to 0.03 Å [19]. The limitations of GPA are that the error in the strain characterization arises while processing amorphous regions or discontinuities at material interfaces and lattices with a strain gradient. Fortunately, Tb-Dy-Fe alloy samples do not contain these characteristics and are suitable for GPA analysis. In this paper, the GPA method was used to calculate the lattice strains and displacement of Tb_0.29_Dy_0.71_Fe_1.95_ material.

**Figure 3:**
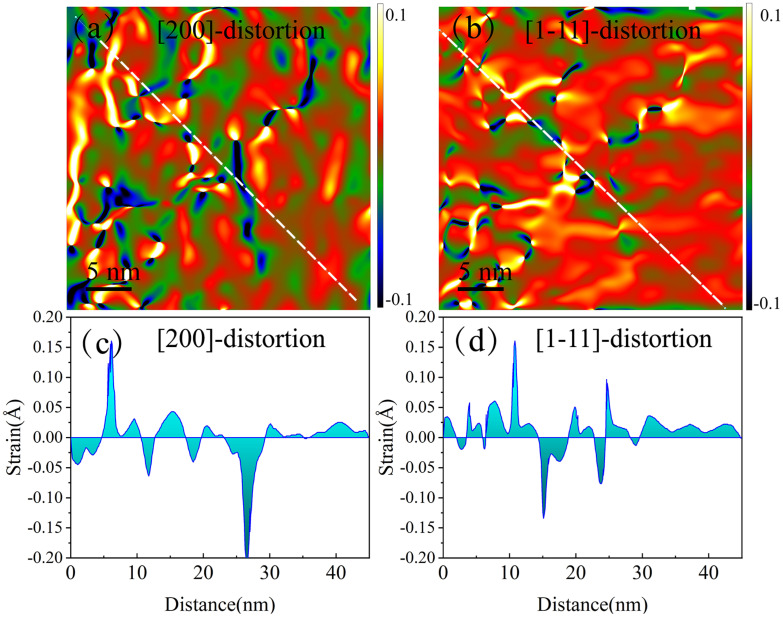
The GPA results of Tb_0.29_Dy_0.71_Fe_1.95_. (**a**,**b**) The distortion maps along the (200) and (1–11) directions obtained using geometric phase analysis (GPA). (**c**,**d**) Distribution of in-plane strain along the white dotted lines in the figures (**a**,**b**).

Taking the *x*-axis parallel to (200) (along the interface) and the *y*-axis parallel to (1–11), the strain fields along the x and y direction can be calculated using GPA. The strain field distribution characterized by GPA along the (200) and (1–11)-FCC directions is shown in Figure 3. Figure 3a,b gives information on the compression (depicted in blue) and expansion (depicted in yellow) of interplanar spacings of (200) and (1–11), respectively. There are regions where compressive strain and expansion strain are concentrated, and also regions where strain is gentle. Here, the tensile and compressive strains are interleaved and irregular. It was found that the compressive or expansion strain region in the strain field in the direction of (200) does not coincide with the regions in the strain field in the direction of (1–11), indicating that the increase or decrease in the distance between (200) crystal planes does not affect the change in the distance between (1–11) crystal planes. The crystal structure does not contract or expand uniformly but has independent disturbances at each lattice parameter. By calculating the distribution of in-plane strain along the line, the strain magnitude can be obtained quantitatively, as shown in Figure 3c,d. The strain variation range of (200) and (1–11) crystal planes is mostly between ±0.17 Å. Moreover, the curves at zero strain are smooth, that is, they are all first-order derivable, indicating that the positive and negative strains are continuously changing, and there is no step-like sudden change.

For the lattice disturbance studied in this paper, it is necessary that atomic column positions are estimated unbiasedly with the highest possible precision. The atomic spacing in STEM images can be read directly by software such as Digital Micrograph, but the accuracy of visual observation is too low. For atomic-resolution STEM images, the intensities of the projected atomic columns peak at the positions of the atomic columns. The exact location of this peak can be obtained using superposition modeling of Gaussian functions [23]. Then the unknown parameters are estimated by fitting this model to the experimental images using a criterion of goodness of fit, quantifying the similarity between the experimental images and the Gaussian mode. For this purpose, the least squares method was used to obtain the optimal solution. This method was combined in a free user-friendly software, StatSTEM, which is freely available under a GNU public license [13]. By using Statstem software, the atomic column position and scattering cross-section in STEM images can be measured with high precision. Quantitative analyses have successfully been applied for two-dimensional (2D) atomic column position measurements with precision in the picometre range from TEM images. The highest attainable precision is reached even for low-dose images. By using this Gaussian model-based fitting method, the atomic column positions can be accurately located, with a resolution orders of magnitude better than the resolution of the electron microscope [13]. In this paper, we used Statstem software to obtain the exact position of each atomic column in the coordinate system. Then, by calculating the distance between the atoms, the precise lattice parameters were obtained.

**Figure 4:**
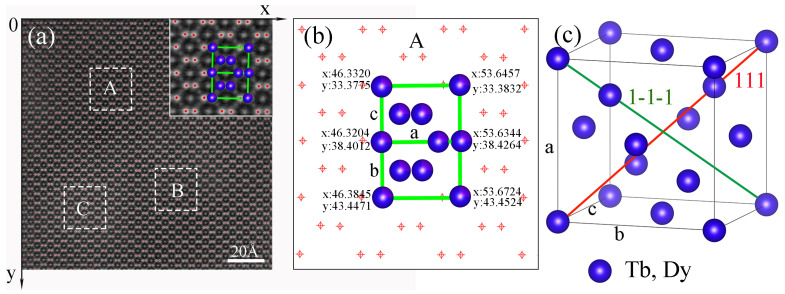
Calculation of the atomic positions by means of Gaussian model-based fitting. (**a**) Coordinate system of atomic positions after Gaussian fitting; (**b**) coordinates of the atoms in region A (white rectangle) and calculated distances between the atoms; and (**c**) corresponding cell model for the crystal structure parameters calculated in (**b**).

Figure 4a shows the result obtained after Gaussian model-based fitting of the image in Figure 2d. In the calculation process, the atomic-resolution photograph is considered the coordinate system. The x- and y-axes are shown in the figure. The positions of the red dots in the figure represent the exact coordinates of each atomic column, and any coordinate value can be directly read from the results. Three typical regions (white rectangles A, B, and C) are selected to calculate the lattice parameters. An enlarged image of region A is shown in Figure 4b. The position relationships between the cell model and atoms are shown in Figure 4c. Therefore, we can use the coordinates to determine the distance between any two atoms. The results of some crystal structure parameters after calculation are shown in Table 1.

Jade5 software (https://materialsdata.com/prodjd.html, accessed on 22 November 2019) was used to calculate the lattice constant of the xrd pattern shown in Figure 1a,b, and the calculated results were both a = 7.33 Å. The lattice constants shown in Table 1 all fluctuate around 7.33 Å. According to Table 1, the crystal structure is not a perfect cubic structure, and the lattice parameters are disturbed based on the cubic structure. The lattice constants have a disturbance on the order of 10^−1^ Å, and L<111> and L<1-1-1> are disturbed within the range of 12.34 Å–12.75 Å. The magnitudes of these disturbances are universal in the sample and are random in size, and there is no step change. Within the same grain, the range of the disturbance of these crystal plane spacings is in the same order of magnitude as that characterized by the XRD peak width. For comparison, the XRD peak width of diamond crystals with similar crystal structures is approximately 0.1 degrees [24]. The lattice constant perturbation range of diamond is 10^−5^ Å, which is much smaller than that of the Tb-Dy-Fe crystal.

The main reason for the broadening of diffraction peaks in directionally solidified samples is obviously the variation in the interplanar crystal spacing. The random perturbation of lattice parameters may be caused by the spontaneous magnetization of magnetic materials and the inhomogeneity of the composition distribution. On the one hand, as we know, the magnetism of magnetic materials originates from the spin magnetic moment of ions. Because the orbital magnetic moment is frozen and does not contribute to the atomic magnetic moment, the atomic magnetic moment μJ in a strong magnetic crystal is spontaneously parallel or antiparallel in a small range (the magnetic domain, called the magnetic order). This magnetic ordering is called spontaneous magnetization. The spontaneous magnetization of rare earth metals and compounds mainly comes from the indirect exchange of electron spins between adjacent atoms (ions), and spontaneous magnetization is generated. The surrounding environment between different atoms is not the same (due to the uneven distribution of components and other reasons), and the exchange between adjacent atoms is also different, so the distance between atoms produces a disturbance phenomenon. On the other hand, Tb and Dy atoms can substitute each other in the crystallographic position, but the distribution of Tb and Dy atoms cannot be completely homogenized even after a long period of heat treatment, and there is still a phenomenon of element segregation. Since the Tb and Dy atoms occupy exactly the same crystallographic position in the RFe_2_ crystal and there is no preferred occupation, the number of Dy atoms adjacent to the Tb atoms at different locations in the sample likely differs. This results in inconsistencies in the lattice constants a, b, and c. The crystal lattice disturbances may also be the reason why Tb-Dy-Fe is more prone to deformation in a magnetic field, which plays an important role in reducing the magnetocrystalline anisotropy to improve the magnetostriction under a low magnetic field.

## 4. Conclusions

We performed STEM studies on Tb_0.29_Dy_0.71_Fe_1.95_ directionally solidified alloy with different magnetic field strengths and employed the GPA method to calculate strain and the Gaussian model-based fitting method to refine the atom positions. The crystal structure of Tb-Dy-Fe alloy is not a standard cubic structure. The lattice constant is disturbed within a small range based on a cubic cell, which is a typical phenomenon in this sample. The range of the disturbance of the crystal lattice is consistent with the results of XRD peak width characterization. This variation in the crystal lattice may be related to the characteristics of the distribution of Tb and Dy atoms in the material.

## Figures and Tables

**Table 1:** Some parameters of the Tb_0.29_Dy_0.71_Fe_1.95_ crystal structure calculated based on the coordinate system.

Crystal Region	a (Å)	b (Å)	c (Å)	L<111> (Å)	L<1-1-1> (Å)
A	7.31	7.11	7.13	12.41	12.47
B	7.30	7.20	7.46	12.65	12.75
C	7.29	7.48	7.11	12.43	12.34

The crystal region refers to the A, B, and C regions in Figure 4a. a, b, and c correspond to the lattice constants in Figure 4. L<111> and L<1-1-1> correspond to the atomic spacings along the diagonals of the cell in Figure 4c.

## Data Availability

Data are contained within the article. The data presented in this study are available in this article.
